# Simplified Gene Knockout by CRISPR-Cas9-Induced Homologous Recombination

**DOI:** 10.1021/acssynbio.1c00194

**Published:** 2021-12-09

**Authors:** Neil C. Dalvie, Timothy Lorgeree, Andrew M. Biedermann, Kerry R. Love, J. Christopher Love

**Affiliations:** † Department of Chemical Engineering, Massachusetts Institute of Technology, Cambridge, Massachusetts 02139, United States; ‡ The Koch Institute for Integrative Cancer Research, Massachusetts Institute of Technology, Cambridge, Massachusetts 02139, United States

**Keywords:** CRISPR, *Pichia pastoris*, gene knockout, DSB repair

## Abstract

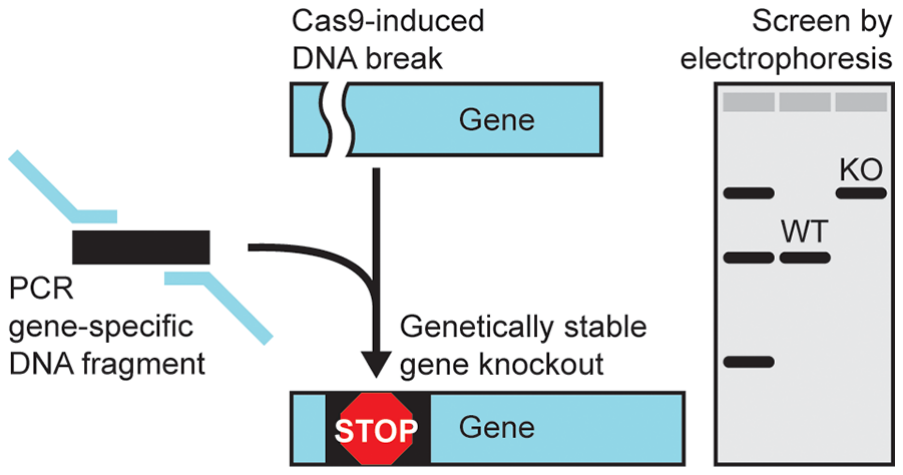

Genetic engineering of industrial cell lines often requires knockout of multiple endogenous genes. Tools like CRISPR-Cas9 have enabled serial or parallelized gene disruption in a wide range of industrial organisms, but common practices for the screening and validation of genome edits are lacking. For gene disruption, DNA repair by homologous recombination offers several advantages over nonhomologous end joining, including more efficient screening for knockout clones and improved genomic stability. Here we designed and characterized a knockout fragment intended to repair Cas9-induced gene disruptions by homologous recombination. We identified knockout clones of *Komagataella phaffii* with high fidelity by PCR, removing the need for Sanger sequencing. Short overlap sequences for homologous recombination (30 bp) enabled the generation of gene-specific knockout fragments by PCR, removing the need for subcloning. Finally, we demonstrated that the genotype conferred by the knockout fragment is stable under common cultivation conditions.

## Introduction

Genetic engineering can help to create and validate industrially useful cell factories.^1^ Disruption, or knockout, is the simplest way to edit endogenous genes because it does not require a cassette for heterologous gene expression or knowledge of the surrounding genomic locus. The recent development of gene editing tools like CRISPR-Cas9 has enabled knockout of genes without selection markers, as exemplified by the multiplexed knockout of 14 genes in Chinese hamster ovary cells to increase the purity of secreted recombinant proteins.^2^ However, screening for knockout genotypes in transformed clones often relies on laborious techniques like sequencing and mass spectrometry, especially for genotypes that do not confer a phenotype that is easily identified by high-throughput screening. Here we describe a method for performing CRISPR-Cas9-mediated gene knockout that can be rapidly screened by DNA electrophoresis, a simple means to affirm a knockout genotype with high fidelity. We also used this approach to generate a knockout genotype that decreased fitness but remained stable after >30 cell doubling times without reversion.

## Results and Discussion

Knockout of genes by CRISPR-Cas9 often occurs through indel mutations.^3^ A single guide RNA (sgRNA) guides Cas9 to a precise location in the host genome, where it creates a double-stranded break (DSB) in the genomic DNA. Cells can survive a DSB when an error-prone repair mechanism like nonhomologous end joining (NHEJ) results in insertion or deletion of one or more base pairs, precluding further binding of the sgRNA. Repairs that result in frameshift mutations can cause an early stop codon in the targeted coding sequence and thereby disrupt gene function. Repair by NHEJ is difficult to predict, so indel mutations must be screened by amplification and Sanger sequencing of the targeted locus to confirm the formation of an early stop codon.^4^ An early stop codon, furthermore, may still allow translation of a truncated protein that retains function, so knockout of protein function may need to be confirmed by evaluation of cellular phenotypes, proteomics, or transcriptomics under relevant environmental conditions. These lengthy screening steps impede rapid iteration of genome edits, especially if the targeted gene does not have a known or an obvious phenotype-altering function.

**Figure 1:**
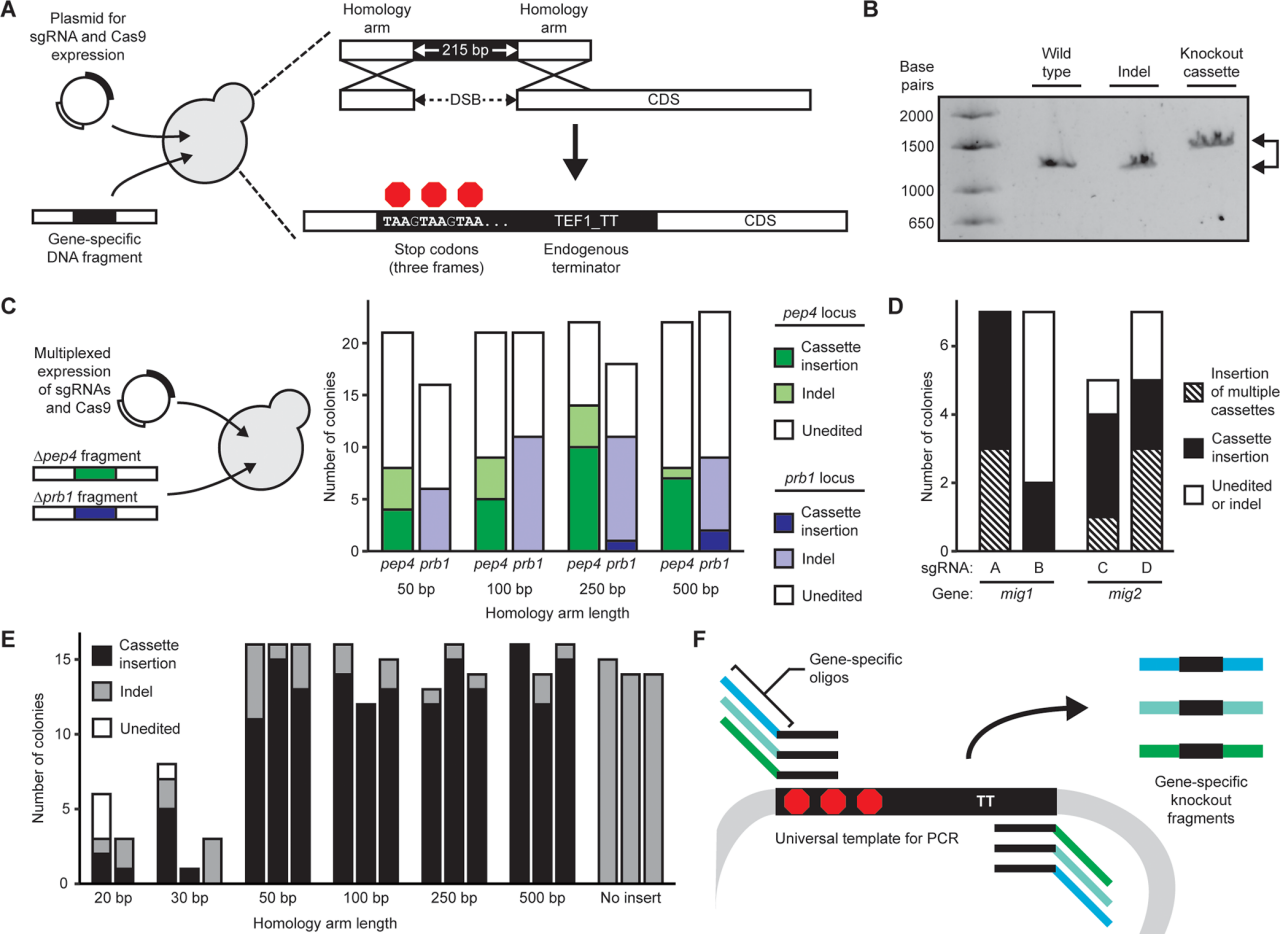
Characterization of a knockout cassette for rapid generation and screening. (A) Schematic of the transformation workflow and integration of the knockout cassette by homologous recombination. (B) Example of DNA gel electrophoresis of knockout fragment integration. PCR was performed on genomic DNA extracted from individual colonies after transformation. (C) Observations from simultaneous targeting of *pep4* and *prb1* with varied knockout fragment homology arm lengths. Each homology arm length represents one transformation. Genomic loci were evaluated by PCR and Sanger sequencing. (D) Integration of the knockout cassette at *mig1* and *mig2* with different sgRNAs. Genomic loci were assessed by PCR and gel electrophoresis. (E) Integration and knockout efficiencies of *gut1* with varied homology arm lengths. Bars represent independent transformations, with all or a maximum of 16 colonies analyzed. (F) Rapid generation of gene-specific knockout fragments by PCR.

We designed a method to facilitate the rapid screening of gene knockouts (Figure 1A). As a case study, we applied this concept in *Komagataella phaffii*, a yeast that is of interest as an alternative host for manufacturing recombinant proteins.^5^ We previously reported a simplified method for CRISPR-Cas9 genome editing in *K. phaffii* using the reporter gene *gut1*.^6^ Here we designed a DNA fragment to enable repair of a DSB in the *gut1* locus by homologous recombination (HR). The fragment comprised three stop codons, one in each reading frame, placed at the 5′ end of the transcription terminator from the *tef1* gene in *K. phaffii*. The fragment was flanked with DNA sequences from the *gut1* locus in *K. phaffii* to enable HR. We transformed the linear knockout fragment along with a plasmid described previously that expresses Cas9 and a sgRNA targeting *gut1*.^6^ We isolated genomic DNA from transformants and amplified the *gut1* coding region by PCR. We analyzed the amplicons by gel electrophoresis and observed a distinct mobility shift in the amplicons from genomic DNA isolated from transformants where the knockout cassette was integrated (Figure 1B). Amplicons from transformants that repaired the DSB with an indel mutation, on the other hand, differed in size by only a few base pairs from colonies with wild-type genotypes. We observed a gel mobility shift in amplicons derived from 122 out of 144 transformants. We confirmed integration of the knockout fragment in all 122 transformants by Sanger sequencing, and 100% of the transformants exhibited deficient growth on glycerol (Table S1). Therefore, gel electrophoresis was a reliable method to identify knockout genotypes with our insertion-based method rapidly.

We previously demonstrated expression of multiple sgRNAs from a single plasmid, which enabled simultaneous editing of multiple genomic loci from a single transformation.^6^ We sought to demonstrate that the knockout fragments reported here are compatible with such multiplexed genome editing. We targeted *pep4* and *prb1*, two protease genes that are commonly disrupted in commercial strains of *K. phaffii*.^7^ We transformed a plasmid that expressed sgRNAs targeting *pep4* and *prb1* along with linear knockout fragments for each gene, with varying lengths of flanking DNA sequence (Figure 1C and Table S2). We observed more integration of the knockout fragments with flanking sequences of 250 or 500 bp than with flanking sequences of 50 or 100 bp. Interestingly, we observed less integration of the knockout fragment into the *prb1* locus than the *pep4* locus. To further assess the effect of genomic locus and sgRNA sequence on knockout fragment integration, we targeted two additional genes, *mig1* and *mig2*, that have been disrupted in efforts to engineer recombinant gene induction in *K. phaffii*. We observed that two different sgRNAs could facilitate integration of the knockout fragment at each genomic locus with different efficiencies (Figures 1D and S1). These results together suggest that the knockout fragment is broadly compatible with many genomic loci but that the rate of integration may depend on the genomic locus. This dependence is consistent with previous observations that DNA repair depends on the local DNA sequence at a genomic locus.^8,9^

We next investigated the length of the flanking DNA sequences needed for efficient HR of the knockout fragment. For this experiment, we selected the *gut1* locus because of its high disruption efficiency. We generated knockout fragments that target *gut1* with flanking sequences between 20 and 500 base pairs of overlap with the desired locus (Figure 1C). Disruption of *gut1* occurred with 100% efficiency for 50–500 bp homology arms. We observed efficient (89.7%) integration of the knockout fragment with homology of 50–500 base pairs, in contrast to prior reports of low HR efficiency in *K. phaffii*.^10,11^ We also observed HR with flanking sequences of 20–30 base pairs, despite fewer transformants overall. Flanking regions as short as 30–50 base pairs could be synthesized from DNA oligo overhangs, allowing gene-specific knockout fragments to be generated by PCR from a universal template (Figure 1D).

**Figure 2:**
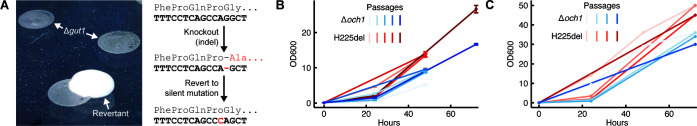
Knockout fragment is stable despite fitness decrease. (A) Characterization of genetic reversion at the *gut1* locus. Cells were grown overnight in YPD medium and stamped onto minimal glycerol agar medium. (B) Growth of engineered strains through serial passaging in 3 mL of plate culture. Error bars represent standard deviations across three biological replicates, passaged individually. (C) Growth of engineered strains through serial passaging in 200 mL flask culture.

After screening transformants for genotype, we left Δ*gut1* transformants on glycerol agar medium for 7–10 days. Interestingly, several Δ*gut1* transformants reverted to a silent in-frame mutation, restoring function of GUT1 (Figure 2A). These revertants occurred only in cells originally observed to have indel mutations. In this study, we obtained 151 transformants that received knockout fragments for *gut1*, *pep4*, and *prb1*. We Sanger-sequenced all 151 transformants and did not observe any restoration of the coding sequence, despite observing in-frame mutations in all three genes in transformants that did not obtain the knockout fragment (Table S2). We therefore hypothesized that integration of the knockout fragment used to screen the transformants may also enhance the genetic stability of a knockout that confers a decrease in fitness, like Δ*gut1* cells growing on glycerol medium.

To further investigate the utility of the knockout fragment, we targeted *och1*, a knockout that is essential for the humanization of glycosylation in *K. phaffii*.^12^ Δ*och1* cells exhibit decreased fitness when cultivated under typical conditions, as manifested by a lower growth rate. We previously constructed a strain containing exogenous *mnn2* and *mns1* and a heterologous reporter peptide K3. With a Δ*och1* genotype, the strain secreted K3 with Man5 N-linked glycosylation.^6^ In the strain with *mnn2*, *mns1*, and *K3*, we targeted *och1* with a knockout fragment, screened the transformants by amplification and gel electrophoresis of the *och1* locus, and isolated a Δ*och1* strain with the knockout fragment integrated. In addition to screening transformants by gel electrophoresis, we screened transformants by visual inspection for the Δ*och1* phenotype, followed by Sanger sequencing of the *och1* locus. Interestingly, we identified one transformant that exhibited morphology similar to a Δ*och1* strain but contained an in-frame mutation (OCH1_H225del; Figure S2A). We evaluated the glycosylation on the K3 reporter peptide from this strain and observed only a small amount of the Man5 glycoform (Figure S2B). This observation exemplifies how frameshift deletion genotypes can be avoided by screening for insertion of the knockout genotypes.

To evaluate the genomic stability of the knockout fragment, we performed repeated growth cycles of both modified strains in 3 mL of plate culture and observed that the Δ*och1* strain grew significantly slower than the OCH1_H225del strain for all growth cycles (paired *t* test, *p* = 0.009; Figure 2B). We observed a similar result when the strains were cultivated in 200 mL flasks (*p* = 0.005; Figure 2C). We performed Sanger sequencing of the *och1* locus at the end of the cultivations and observed unaltered genotypes for both strains. These cultivations indicate that the Δ*och1* strain is less fit than the OCH1_H225del strain under these growth conditions. Despite this decrease in fitness, the Δ*och1* genotype conferred by the knockout fragment was stable after >10^12^ cell divisions.

## Conclusion

We have described here a strategy for rapidly generating gene knockouts. The reported knockout fragment is stable under common cultivation conditions and avoids the potential for in-frame mutations and genetic reversion. Suppressor genotypes would, in theory, require precise mutations between each of three stop codons and excision of the transcriptional terminator. In addition, screening for knockouts by gel electrophoresis enables rapid iteration of gene knockouts without the need to wait 1–2 days for Sanger sequencing of genomic loci. We demonstrated integration of the knockout fragment at six genomic loci and hypothesize that the integration efficiency is dependent on the locus. We also demonstrated HR of the knockout fragment with flanking sequences of <50 bp. This method enables generation of gene-specific knockout fragments by PCR with unique primers, saving an additional 1–2 days of DNA synthesis or cloning. HR can also enable flexibility in gene knockout: flanking sequences could be designed to delete entire sequences or genes from the genome. Here our knockout fragment was based on an endogenous terminator, but in principle, the fragment could include exogenous genes, custom expression sequences, or DNA barcodes at engineered loci.

## Materials and Methods

### Yeast Strains and Vectors

PCR was performed using Q5 Hotstart High Fidelity Master Mix (NEB) according to the manufacturer’s instructions. Fragment assembly was performed using HiFi Assembly Master Mix (NEB) according to the manufacturer’s instructions. Plasmids were stored and propagated in DH5alpha *Escherichia coli* (NEB). Primer synthesis and Sanger sequencing were performed by Genewiz Inc. Vectors for expression of Cas9 and sgRNAs were constructed as described previously.^6^ All strains were derived from wild-type *K. phaffii* (NRRL Y-11430). Glycosylation-engineered strains were constructed previously.^6^

### Transformation of the Knockout Fragment

The knockout fragment template was obtained by amplification from the *K. phaffii* genome (Table S3). Disruption of *gut1*, *mig1*, *mig2*, and *och1* was performed by electroporation of *K. phaffii* with 100 ng of the Cas9/sgRNA plasmid and 1 μg of linear knockout fragment DNA. Multiplexed disruption of *pep4* and *prb1* was performed by electroporation of *K. phaffii* with 100 ng of the Cas9/sgRNA plasmid and 1 pmol of each linear knockout fragment DNA. Knockout efficiency was assessed by random selection of 8–24 transformants from each transformation, with no phenotypic selection. Genotype was assessed by PCR of the targeted locus followed by gel electrophoresis or Sanger sequencing. *Gut1* phenotype was assessed after selection of random colonies, as described previously.^6^ Transformants with an *Δoch1* genotype typically appeared 1–2 weeks after wild-type transformants.

### Cultivations for Growth Measurements

Growth studies were performed in 3 mL of culture in 24-well deep-well plates (25 °C, 600 rpm) or 200 mL of culture in 1 L baffled shake flasks (25 °C, 250 rpm). Cells were cultivated in complex medium (potassium phosphate buffer, pH 6.5, 1.34% nitrogen base without amino acids, 1% yeast extract, 2% peptone). Cells were inoculated at an optical density at 600 nm (OD_600_) of 0.1 and grown with 4% glycerol feed. After 2–3 days for each cycle, cells were pelleted and inoculated into fresh medium at OD_600_ = 0.1.

### Analysis of K3 Glycosylation from the OCH1_H255del Strain

Cells were grown in 3 mL of culture in 24-well deep-well plates in complex medium as described above. Cells were inoculated at OD_600_ = 0.1, outgrown for 24 h with 4% glycerol feed, pelleted, and resuspended in fresh medium with 3% methanol to induce recombinant gene expression. Supernatant samples were collected after 24 h of production. K3 protein was purified as described previously.^6^ Intact mass spectrometry was performed as described previously.^13^

## Supporting Information Available

Tabular results of knockout screening and multiplexed knockout screening, characterization of a Δ*och1* knockout suppressor mutation, sequences of Cas9/sgRNA editing plasmids, and sequences of knockout fragments (ZIP)
