# Supplementary material for: Simplified Gene Knockout by CRISPR-Cas9-Induced Homologous Recombination
Source: ACS Synth Biol. 2021 Dec 9;11(1):497–501. doi: 10.1021/acssynbio.1c00194 (PMC8787811; doi:10.1021/acssynbio.1c00194)
Supplement: Supplementary file 1 — sb1c00194_si_001.zip [file sb1c00194_si_001.zip › Supporting Information.docx]

**Supporting Information**

**Simplified gene knockout by CRISPR-Cas9 induced homologous recombination**

Neil C. Dalvie^1,2^, Timothy Lorgeree^1,2^, Andrew M. Biedermann^1,2^, Kerry R. Love^1,2^, J. Christopher Love^1,2^*

^1^Department of Chemical Engineering, Massachusetts Institute of Technology, Cambridge, Massachusetts 02139, United States

^2^The Koch Institute for Integrative Cancer Research, Massachusetts Institute of Technology, Cambridge, Massachusetts 01239, United States

*Correspondence to: [clove@mit.edu](mailto:clove@mit.edu)


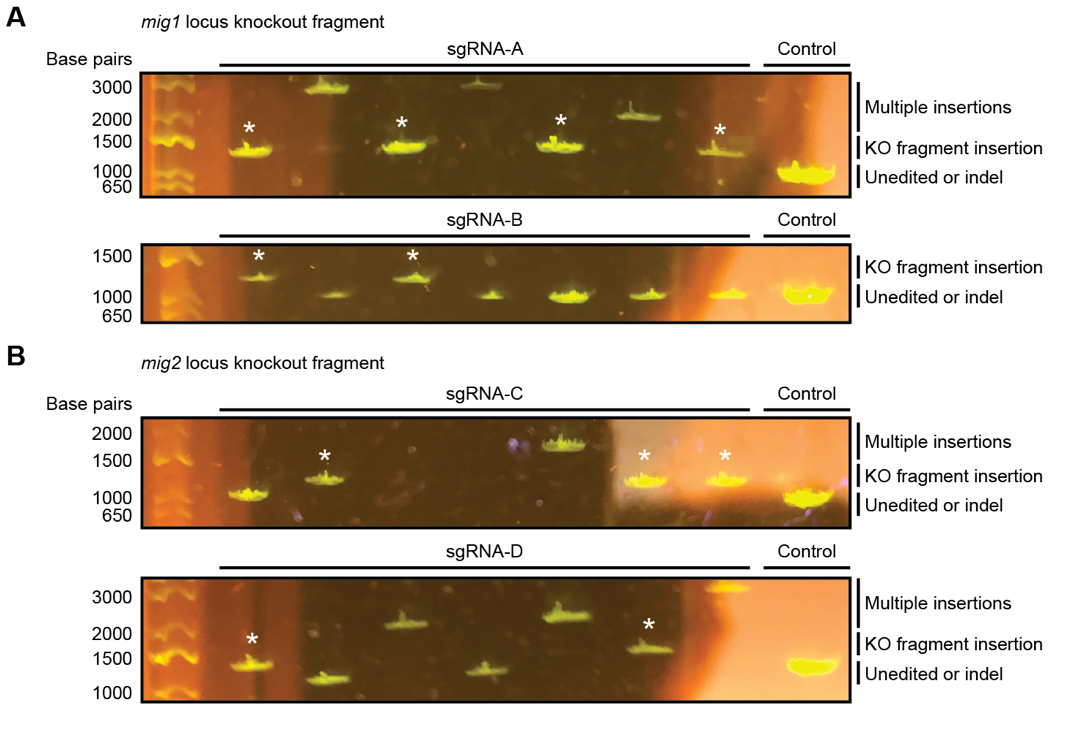


Figure S1. DNA gel electrophoresis of the (A) *mig1* and (B) *mig2* loci to screen for knockout fragment insertion.


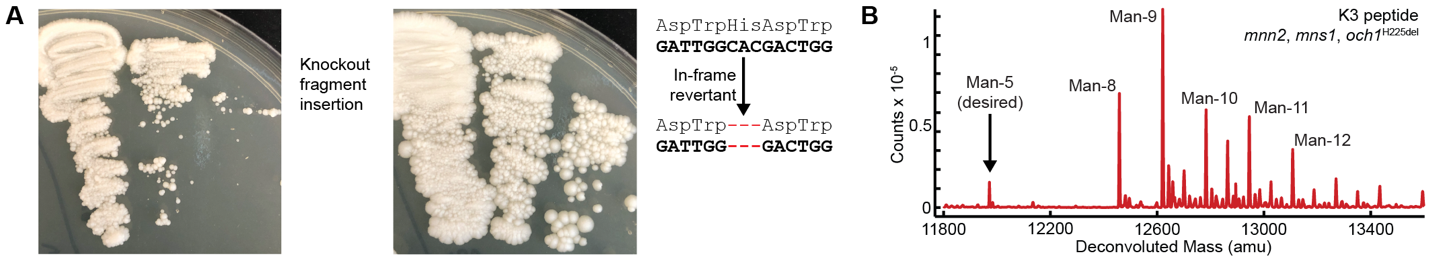


Figure S2. Characterization of an in-frame mutation in *och1*

A) Comparison of cell morphology of Δ*och1* and OCH1_H225del strains. B) Intact mass spectroscopy of the reporter peptide K3 from the OCH1_H225del strain.

Table S1. Genotype of *gut1* and phenotype of transformant colonies

| Replicate | Homology arm length (bp) | Sanger sequencing observation | Gel electrophoresis observation | GUT1 phenotype (growth on glycerol) |
| --- | --- | --- | --- | --- |
| A | 100 | Knockout fragment | Shift | No growth |
| A | 100 | Knockout fragment | Shift | No growth |
| A | 100 | Indel | WT | No growth |
| A | 100 | Knockout fragment | Shift | No growth |
| A | 100 | Knockout fragment | Shift | No growth |
| A | 100 | Knockout fragment | Shift | No growth |
| A | 100 | Knockout fragment | Shift | No growth |
| A | 100 | Knockout fragment | Shift | No growth |
| A | 100 | Knockout fragment | Shift | No growth |
| A | 100 | Knockout fragment | Double | No growth |
| A | 100 | Knockout fragment | Shift | No growth |
| A | 100 | Knockout fragment | Shift | No growth |
| A | 100 | Knockout fragment | Shift | No growth |
| A | 100 | Knockout fragment | Shift | No growth |
| A | 100 | Knockout fragment | Shift | No growth |
| A | 100 | Indel | WT | No growth |
| B | 100 | Knockout fragment | Shift | No growth |
| B | 100 | Knockout fragment | Shift | No growth |
| B | 100 | No data | No data | No growth |
| B | 100 | No data | No data | No growth |
| B | 100 | No data | No data | No growth |
| B | 100 | Knockout fragment | Shift | No growth |
| B | 100 | Knockout fragment | Shift | No growth |
| B | 100 | Knockout fragment | Shift | No growth |
| B | 100 | Knockout fragment | No data | No growth |
| B | 100 | Knockout fragment | Shift | No growth |
| B | 100 | No data | No data | No growth |
| B | 100 | Knockout fragment | Shift | No growth |
| B | 100 | Knockout fragment | Shift | No growth |
| B | 100 | Knockout fragment | Shift | No growth |
| B | 100 | Knockout fragment | Shift | No growth |
| B | 100 | Knockout fragment | Shift | No growth |
| C | 100 | Indel | WT | No growth |
| C | 100 | Knockout fragment | Shift | No growth |
| C | 100 | Knockout fragment | Shift | No growth |
| C | 100 | Knockout fragment | Shift | No growth |
| C | 100 | Knockout fragment | Shift | No growth |
| C | 100 | Knockout fragment | Shift | No growth |
| C | 100 | Knockout fragment | Shift | No growth |
| C | 100 | Knockout fragment | Shift | No growth |
| C | 100 | Knockout fragment | Shift | No growth |
| C | 100 | No data | No data | No growth |
| C | 100 | Knockout fragment | Shift | No growth |
| C | 100 | Knockout fragment | Shift | No growth |
| C | 100 | Indel | WT | No growth |
| C | 100 | Knockout fragment | Shift | No growth |
| C | 100 | Knockout fragment | Shift | No growth |
| C | 100 | Knockout fragment | Shift | No growth |
| A | 250 | Knockout fragment | Shift | No growth |
| A | 250 | Knockout fragment | Shift | No growth |
| A | 250 | Knockout fragment | Shift | No growth |
| A | 250 | Knockout fragment | Shift | No growth |
| A | 250 | Knockout fragment | Shift | No growth |
| A | 250 | Knockout fragment | Shift | No growth |
| A | 250 | Indel | WT | No growth |
| A | 250 | No data | No data | No growth |
| A | 250 | Knockout fragment | Shift | No growth |
| A | 250 | No data | No data | No growth |
| A | 250 | Knockout fragment | Shift | No growth |
| A | 250 | Knockout fragment | Shift | No growth |
| A | 250 | Knockout fragment | Shift | No growth |
| A | 250 | Knockout fragment | Shift | No growth |
| A | 250 | No data | No data | No growth |
| A | 250 | Knockout fragment | Shift | No growth |
| B | 250 | Knockout fragment | Shift | No growth |
| B | 250 | Knockout fragment | Shift | No growth |
| B | 250 | Knockout fragment | Shift | No growth |
| B | 250 | Knockout fragment | Shift | No growth |
| B | 250 | Knockout fragment | No data | No growth |
| B | 250 | Knockout fragment | Shift | No growth |
| B | 250 | Knockout fragment | Shift | No growth |
| B | 250 | Knockout fragment | Shift | No growth |
| B | 250 | Knockout fragment | Shift | No growth |
| B | 250 | Knockout fragment | Shift | No growth |
| B | 250 | Knockout fragment | Shift | No growth |
| B | 250 | Indel | WT | No growth |
| B | 250 | Knockout fragment | Shift | No growth |
| B | 250 | Knockout fragment | Shift | No growth |
| B | 250 | Knockout fragment | Shift | No growth |
| B | 250 | Knockout fragment | Shift | No growth |
| C | 250 | Knockout fragment | Shift | No growth |
| C | 250 | Knockout fragment | Shift | No growth |
| C | 250 | No data | No data | No growth |
| C | 250 | Knockout fragment | Shift | No growth |
| C | 250 | Knockout fragment | Shift | No growth |
| C | 250 | Knockout fragment | Shift | No growth |
| C | 250 | Knockout fragment | Shift | No growth |
| C | 250 | Knockout fragment | Shift | No growth |
| C | 250 | No data | No data | No growth |
| C | 250 | Indel | WT | No growth |
| C | 250 | Knockout fragment | Shift | No growth |
| C | 250 | Knockout fragment | Shift | No growth |
| C | 250 | Knockout fragment | Shift | No growth |
| C | 250 | Knockout fragment | Shift | No growth |
| C | 250 | Knockout fragment | Shift | No growth |
| C | 250 | Knockout fragment | Shift | No growth |
| A | 500 | Knockout fragment | Shift | No growth |
| A | 500 | Knockout fragment | Shift | No growth |
| A | 500 | Knockout fragment | Shift | No growth |
| A | 500 | Knockout fragment | Shift | No growth |
| A | 500 | Knockout fragment | Shift | No growth |
| A | 500 | Knockout fragment | Shift | No growth |
| A | 500 | Knockout fragment | Shift | No growth |
| A | 500 | Knockout fragment | Shift | No growth |
| A | 500 | Knockout fragment | Shift | No growth |
| A | 500 | Knockout fragment | Shift | No growth |
| A | 500 | Knockout fragment | Shift | No growth |
| A | 500 | Knockout fragment | Shift | No growth |
| A | 500 | Knockout fragment | Shift | No growth |
| A | 500 | Knockout fragment | Shift | No growth |
| A | 500 | Knockout fragment | Shift | No growth |
| A | 500 | Knockout fragment | Shift | No growth |
| B | 500 | Knockout fragment | Shift | No growth |
| B | 500 | Knockout fragment | Shift | No growth |
| B | 500 | Indel | WT | No growth |
| B | 500 | Knockout fragment | Shift | No growth |
| B | 500 | Knockout fragment | Shift | No growth |
| B | 500 | Knockout fragment | Shift | No growth |
| B | 500 | Knockout fragment | Shift | No growth |
| B | 500 | Knockout fragment | Shift | No growth |
| B | 500 | Knockout fragment | No data | No growth |
| B | 500 | Knockout fragment | Shift | No growth |
| B | 500 | No data | No data | No growth |
| B | 500 | Indel | WT | No growth |
| B | 500 | No data | No data | No growth |
| B | 500 | Knockout fragment | Shift | No growth |
| B | 500 | Knockout fragment | Shift | No growth |
| B | 500 | Knockout fragment | No data | No growth |
| C | 500 | Knockout fragment | Shift | No growth |
| C | 500 | Knockout fragment | Shift | No growth |
| C | 500 | Knockout fragment | Shift | No growth |
| C | 500 | Knockout fragment | Shift | No growth |
| C | 500 | Indel | WT | No growth |
| C | 500 | Knockout fragment | Shift | No growth |
| C | 500 | Knockout fragment | Shift | No growth |
| C | 500 | Knockout fragment | Shift | No growth |
| C | 500 | Knockout fragment | Shift | No growth |
| C | 500 | Knockout fragment | Shift | No growth |
| C | 500 | Knockout fragment | Shift | No growth |
| C | 500 | Knockout fragment | Shift | No growth |
| C | 500 | Knockout fragment | Shift | No growth |
| C | 500 | Knockout fragment | Shift | No growth |
| C | 500 | Knockout fragment | Double | No growth |
| C | 500 | Knockout fragment | Shift | No growth |
| A | No ins | Indel | WT | No growth |
| A | No ins | Indel | WT | No growth |
| A | No ins | Indel | WT | No growth |
| A | No ins | Indel | WT | No growth |
| A | No ins | Indel | WT | No growth |
| A | No ins | Indel | WT | No growth |
| A | No ins | Indel | WT | No growth |
| A | No ins | Indel | WT | No growth |
| A | No ins | No data | No data | No growth |
| A | No ins | Indel | WT | No growth |
| A | No ins | Indel | WT | No growth |
| A | No ins | Indel | WT | No growth |
| A | No ins | Indel | WT | No growth |
| A | No ins | Indel | WT | No growth |
| A | No ins | Indel | WT | No growth |
| A | No ins | Indel | WT | No growth |
| B | No ins | Indel | WT | No growth |
| B | No ins | No data | No data | No growth |
| B | No ins | Indel | WT | No growth |
| B | No ins | Indel | WT | No growth |
| B | No ins | Indel | WT | No growth |
| B | No ins | Indel | WT | No growth |
| B | No ins | Indel | No data | No growth |
| B | No ins | Indel | WT | No growth |
| B | No ins | No data | WT | No growth |
| B | No ins | Indel | WT | No growth |
| B | No ins | Indel | WT | No growth |
| B | No ins | Indel | WT | No growth |
| B | No ins | No data | No data | No growth |
| B | No ins | Indel | WT | No growth |
| B | No ins | Indel | WT | No growth |
| B | No ins | Indel | WT | No growth |
| C | No ins | Indel | WT | No growth |
| C | No ins | Indel | No data | WT |
| C | No ins | Indel | WT | No growth |
| C | No ins | Indel | WT | No growth |
| C | No ins | No data | WT | No growth |
| C | No ins | Indel | WT | No growth |
| C | No ins | Indel | WT | No growth |
| C | No ins | Indel | WT | No growth |
| C | No ins | Indel | WT | No growth |
| C | No ins | Indel | WT | No growth |
| C | No ins | No data | WT | No growth |
| C | No ins | Indel | WT | No growth |
| C | No ins | Indel | WT | No growth |
| C | No ins | Indel | WT | No growth |
| C | No ins | Indel | WT | No growth |
| C | No ins | Indel | WT | No growth |

Table S2. Multiplexed knockout of *pep4* and *prb1*

| **50 bp homology arms** | |  | **100 bp homology arms** | |  | **250 bp homology arms** | |  | **500 bp homology arms** | |
| --- | --- | --- | --- | --- | --- | --- | --- | --- | --- | --- |
| ***pep4*** | ***prb1*** |  | ***pep4*** | ***prb1*** |  | ***pep4*** | ***prb1*** |  | ***pep4*** | ***prb1*** |
| KF | Indel |  | KF | Indel |  | KF | Indel |  | KF | KF |
| KF | Indel |  | KF | no data |  | KF | Indel |  | KF | Indel |
| KF | no data |  | KF | In-frame |  | KF | Indel |  | KF | Indel |
| KF | Indel |  | KF | Indel |  | KF | Indel |  | KF | Indel |
| In-frame | Indel |  | KF | Indel |  | KF | Indel |  | KF | Indel |
| In-frame | no data |  | Indel | Indel |  | KF | Indel |  | KF | no data |
| Indel | no data |  | Indel | Indel |  | KF | no data |  | KF | WT |
| Indel | no data |  | Indel | Indel |  | KF | no data |  | Indel | Indel |
| no data | no data |  | Indel | no data |  | KF | no data |  | no data | KF |
| no data | no data |  | no data | Indel |  | KF | Indel |  | no data | Indel |
| no data | no data |  | no data | Indel |  | Indel | Indel |  | WT | Indel |
| WT | Indel |  | no data | no data |  | Indel | Indel |  | WT | WT |
| WT | Indel |  | WT | Indel |  | Indel | no data |  | WT | WT |
| WT | no data |  | WT | Indel |  | Indel | WT |  | WT | WT |
| WT | WT |  | WT | WT |  | no data | KF |  | WT | WT |
| WT | WT |  | WT | WT |  | no data | no data |  | WT | WT |
| WT | WT |  | WT | WT |  | WT | Indel |  | WT | WT |
| WT | WT |  | WT | WT |  | WT | no data |  | WT | WT |
| WT | WT |  | WT | WT |  | WT | WT |  | WT | WT |
| WT | WT |  | WT | WT |  | WT | WT |  | WT | WT |
| WT | WT |  | WT | WT |  | WT | WT |  | WT | WT |
| WT | WT |  | WT | WT |  | WT | WT |  | WT | WT |
| WT | WT |  | WT | WT |  | WT | WT |  | WT | WT |
| WT | WT |  | WT | WT |  | WT | WT |  | WT | WT |

| Knockout fragment insertion = KF |
| --- |
| Indel mutation causing frameshift |
| In-frame deletion |
| No editing = WT |
| No PCR product = no data |

Table S3. Knockout fragment sequence.

TAAGTAAGTAAATTGCTTGAAGCTTTAATTTATTTTATTAACATAATAATAATACAAGCATGATATATTTGTATTTTGTTCGTTAACATTGATGTTTTCTTCATTTACTGTTATTGTTTGTAACTTTGATCGATTTATCTTTTCTACTTTACTGTAATATGGCTGGCGGGTGAGCCTTGAACTCCCTGTATTACTTTACCTTGCTATTACTTAAT

Table S4. List of appended sequence files

| Sequence type |  |
| --- | --- |
| Cas9 and sgRNA expression plasmid | *gut1* sgRNA |
|  | *mig1* sgRNA-A |
|  | *mig1* sgRNA-B |
|  | *mig2* sgRNA-C |
|  | *mig2* sgRNA-D |
|  | *och1* sgRNA |
|  | *pep4* and *prb1* multiplexed sgRNAs |
| Knockout fragment for homologous recombination repair | *gut1* |
|  | *mig1-*A |
|  | *mig1-*B |
|  | *mig2-*C |
|  | *mig2-*D |
|  | *och1* |
|  | *pep4* |
|  | *prb1* |
